# Crossing the Rubicon: Behaviorism, Language, and Evolutionary Continuity

**DOI:** 10.3389/fpsyg.2020.00653

**Published:** 2020-04-21

**Authors:** Michael C. Corballis

**Affiliations:** Faculty of Science, School of Psychology, The University of Auckland, Auckland, New Zealand

**Keywords:** behaviorism, cognition, evolution, imagination, language

## Abstract

Euan Macphail’s work and ideas captured a pivotal time in the late 20th century when behavioral laws were considered to apply equally across vertebrates, implying equal intelligence, but it was also a time when behaviorism was challenged by the view that language was unique to humans, and bestowed a superior mental status. Subsequent work suggests greater continuity between humans and their forebears, challenging the Chomskyan assumption that language evolved in a single step (“the great leap forward”) in humans. Language is now understood to be based on an amalgam of cognitive functions, including mental time travel, theory of mind, and what may be more broadly defined as imagination. These functions probably evolved gradually in hominin evolution and are present in varying degrees in non-human species. The blending of language into cognition provides for both interspecies differences in mental function, and continuity between humans and other species. What does seem to be special to humans is the ability to communicate the contents of imagination, although even this is not absolute, and is perhaps less adaptive than we like to think.

## Introduction

Language is our Rubicon, and no brute will dare cross it. — Müller (1861, p. 403)

In the days when behaviorism was the dominant paradigm in psychology, it was widely assumed not to matter which species you studied. In the late 1950s, when I first began to study psychology, rats were the species of choice, and a few years later they were more or less seamlessly replaced by pigeons. There was perhaps a limit as to how far one could go along the branches of the evolutionary tree, and in the 1950s the comparative psychologist James V. McConnell made a semi-serious attempt to introduce worms. *The Worm Runners’ Digest* was established in 1959 and rather surprisingly lasted 20 years, mainly as a stimulus for countless school projects, and research reports often designed for humor rather than serious science.

With one exception, vertebrates were assumed to conform to the same universal behavioral laws. The British psychologist Euan Macphail went so far as to suggest that all were of equal intelligence, and for behaviorists this left little room for comparative psychology, or for ethology, which was left to the zoologists. Know one species, and you know them all, at least as far as intellect was concerned. That one exception, though, was *Homo sapiens*, blessed with the faculty of language, raising human intelligence to a different level. Macphail held this position for over 20 years:

It may be ironic that we suppose (e.g., Macphail, 1982) that the key to our intelligence lies in the appearance in humans alone of the capacity for language, and that the exploitation of linguistic skills (and other related symbolic skills, such as those of mathematics) results in our unique intelligence (Macphail and Bolhuis, 2001, p. 361).

This continued a tradition from at least Biblical times, and extending through most of western philosophy, of placing humans on a pedestal, somewhere between apes and angels. Hobbes (1651), in his essay *Of Man*, wrote that “the first author of speech was God himself,” and the 17th-century philosopher Descartes (1984) supposed that language was the property that elevated humans above other animals, and bestowed the capacity for free will. Müller (1861) was Professor of Philology at Oxford University, and the quote that opened this article was his response to Darwin’s (1859) book *On the Origin of Species*.

Skinner (1957) had been aware of the challenge posed by language, and made a monumental effort to account for language in behavioral terms in his book *Verbal Behavior*. Unfortunately, it was published in the same year as *Syntactic Structures* by a young Noam Chomsky (1957), who was part of a new generation launching what came to be called the “cognitive revolution.” This was followed by Chomsky’s (1959) influential but damning review of Skinner’s book, which to many signaled the end of behaviorism as the dominant paradigm of psychology. Rats and pigeons were replaced by undergraduate students, eager for course credit, being tested for cognitive skills, and tapping at keys with the same dedication as their pigeon forebears.

The cognitive revolution also shifted attention away from learning toward structures generally assumed to be innate. In spite of the fact that some 7,000 different languages exist in the world, each incomprehensible to nearly all of the others, language was taken to depend on an innate endowment, and differing from animal communication in that it was based on computational rules applying universally but exclusively to humans. These rules were once called “deep structure” but later “universal grammar” (Chomsky, 1995). Universal grammar is considered a recursive system permitting an infinite variety of utterances, or “the infinite use of finite means” (von Humboldt, 1836/1999). Chomsky proposed, moreover, that universal grammar emerged fully-fledged in our own species in a single step, the “great leap forward,” and perhaps even in a single individual, whom Chomsky (2010) whimsically named Prometheus. This runs counter to evolutionary theory; as Darwin (1859) himself put it, “natural selection can act only by taking advantage of slight successive variations; she can never take a leap, but must advance by the shortest and slowest steps” (p. 194).

At the end of the second decade of the 21st century, we are perhaps moving away from the Chomskyan era and the rigid insistence on innate structures underlying cognition. Given the often vast differences between languages, the very concept of universal grammar has been disputed. Evans and Levinson (2009), for example, concluded that “the emperor of Universal Grammar has no clothes” (p. 438) and Tomasello (2009) remarked similarly that “Universal grammar is dead” (p. 470). Some languages, such as those of the Pirahä of Brazil (Everett, 2005) or the Iatmul of New Guinea (Evans, 2009), do not seem to display the recursive structure of universal grammar. Viewed as a communication system, at least, language seems not to have a universal basis.

## Language as Thought

These objections have been partly finessed by Chomsky himself, who has insisted that universal grammar is part of what he calls *I-language*, which is not fundamentally concerned with communicative language at all. Rather, it is a component of thought. Actual languages, whether spoken, signed, or written, are part of a process of *externalization*, the transforming of internal thoughts into communicable form. This seems partly to resolve the problem of why there are so many languages in the world, and why they are so diverse. As Chomsky (2015b) put it: “It is a familiar fact (*sic*) that that the complexity and variety of language appears to be localized overwhelmingly—and perhaps completely—in externalization (p. xi).” This renders actual languages peripheral to the understanding of universal grammar itself.

The idea that the essence of language lies in thought rather than in communication should not be taken to mean that we think in words, as many have long claimed. Plato, for example, through the mouth of Sophocles, wrote that “the soul when thinking appears to me to be just talking—asking questions of herself and answering them” (Jowett, 1892, p. 190), and in 1798 Immanuel Kant wrote that “Thinking is speaking to ourselves” (quoted in translation by Butts, 1988, p. 278). The founder of behaviorism, John B. Watson, similarly equated thought with subvocal speech (Watson, 1928). For Chomsky, though, thought is primary, and words merely provide the means for articulating those thoughts, in agreement with the 17th century philosopher John Locke (1690/2017):

We should have a great many fewer disputes in the world if words were taken for what they are, the signs of our ideas only, and not for the things themselves (p. 185).

Although the structure of I-language is not easily discerned in the thickets of languages themselves, Chomsky’s views have become progressively simplified, culminating in *The Minimalist Program* (Chomsky, 1995, 2015b), which seemingly reduces the gap between humans and other species. Universal grammar is reduced to a single operation called *Unbounded Merge* (or simply *Merge*) in which elements are combined in recursive fashion to form new entities, which can themselves be merged, and so on. It is the basic mechanism for the construction of hierarchies, with language as the most overt example, but there are other examples, as we shall see later. In Chomsky’s view, Merge applies not directly to communicative languages themselves, but rather to the abstract elements of I-language. Nevertheless, the operations of Merge might be manifest indirectly in spoken language, say, as successive merges of phonemes to form syllables, syllables to form words, words to form phrases, phrases to form sentences, and so on. But Merge itself seems a simple operation, leading Berwick and Chomsky (2016) to remark that, “we simply don’t have as much to explain, reducing the Darwinian paradox” (p. 11). These authors nevertheless continue to insist that universal grammar is unique to humans, as reflected in the very title of their book, *Why Only Us?*

These developments blur the distinctions proposed by Macphail. Language may not after all be independent of animal intelligence, or of thought itself. Chomsky’s Merge is presented as a highly specialized process, unique to humans, but generative processing may actually be a general aspect of thought, and evident in non-human animals, as I suggest later. One example is what has been termed *mental time travel*, the capacity to “replay” past events or imagine future ones (Suddendorf and Corballis, 1997, 2007)—also dubbed *episodic memory* and *episodic projection*, respectively. Indeed mental time travel may capture at least some of the properties of Merge itself, combining various elements into episodes, and accounting for much of the generativity of language itself. It underlies the linguistic property of *displacement*, defined by Hockett (1960) as the ability “to talk about things that are remote in space or time (or both) from where the talking is going on.” Expressive language may have evolved precisely to enable us to communicate about the non-present (Corballis, 2009, 2017; Gärdenfors and Osvath, 2010; Bickerton, 2014).

One brain structure critically involved in mental time travel is the hippocampus. In one of our own brain-imaging studies (Martin et al., 2011), participants were asked to describe 110 episodes in their lives, and then construct 110 possible future episodes based on scrambled aspects of the remembered ones—tasks seemingly easily accomplished. While recalling or imagining these events, the hippocampus was active—more anteriorly for future events than past ones. The hippocampus also features in animal studies of mental time travel, discussed below.

The idea of mental time travel can be broadened to encompass imagination, which can include purely imaginary events and stories that are not necessarily grounded in reality. Indeed episodic projection necessarily includes the imagining of events that have not already occurred, and even episodic memories are often distortions of what actually occurred in the past, or are fabrications. We delight in making things up. Language, then, may be the externalization of imagination, involving richer and more experience-based constructions than suggested by the concept of Merge (Corballis, 2017). Along the same lines, Dor (2015) describes language as “the instruction of imagination.”

Zuberbühler (2019) suggests that grammar itself derives from the perceived structure of events and evolved before communicative language itself. This compositional structure is made up of components such as actors, agents, patients, predication, and so on, and marking them with communicative signals. These components are merged in multiple ways to make up the real or imagined events, or episodes, of our lives. The linguist Paul Deane (1992) earlier argued that grammar is based on our understanding of space, and what happens in it. The way in which experience is structured in space and time may well be universal, and provide a basis for Chomsky’s universal grammar. We all live in a spatiotemporal world, inhabited by things, people and artifacts of our own making. As humans, we are similarly size-scaled, differing from ants or elephants. We view the world at roughly the same angle and move about at roughly the same speed—at least until fast cars and airplanes disturbed our leisured pace. The mind no doubt adapted to these kinds of parameters, creating a near-universality of understanding. As I summarized earlier: “What Chomsky called universal grammar may therefore depend more on how long we and our forebears have inhabited the world and reacted to it than on some new internal program called unbounded Merge” (Corballis, 2017, p. 190).

But it is more than physical. We have also evolved as social animals, understanding how people function. This includes is *theory of mind*, which underlies the capacity to attribute mental states to other. This is recursive: I may know that you know something (level 1 theory of mind), but also know that you know that I know this (level 2). We can proceed, perhaps with effort, to higher levels of recursion, often in the interests of Machiavellian intrigue. Dunbar (2004) suggests level 5 recursion underlies the mutual understanding of gods: “I suppose that you think that I believe there are gods who intend to influence our future because they understand our desires (p. 185).” Grice (1989) proposed that at least level 2 theory of mind is necessary for communicative language:

He said that P: he could not have done this unless he thought that Q; he knows (and knows that I know that he knows) that I will realize that it is necessary to suppose that Q; he has done nothing to stop me thinking that Q; so he intends me to think, or is at least willing for me to think that Q (pp. 30–31).

Or to put it in more everyday terms, communicative language requires that the speaker knows what’s in the recipient’s mind, and knows that the recipient knows that she know this!

The importance of theory of mind to natural language is elaborated by Sperber and Wilson (2002) and by Scott-Phillips (2015), who show that mutual understanding between speakers can often reduce the need for words themselves. As Scott-Phillips puts it, language is *underdetermined*. Even Chomsky (2011) seems to agree. He writes: “Communication relies on largely shared cognoscitive powers, and succeeds insofar as similar mental constructs, background, concerns, and presuppositions allow for similar perspectives to be reached” (p. 10). He does go on, however, to assert that these features are not present in animal communication.

Friederici (2019) raises the question of whether there is a single network in the brain underlying hierarchy processing and concludes, based on brain imaging, that there is not. The hierarchical aspect of language appears to be grounded neurologically in Area 44, part of Broca’s area, in the left hemisphere. Theory of mind, in contrast, seems to depend on a bilateral fiber tract involving temporal and parietal lobes and anterior parts of the dorsal fiber tract, but does not include Broca’s area. The harmonic and melodic structure of music are also hierarchical in structure, and also seem to activate Broca’s area, along with its right-hemisphere homolog. Mathematics is also structured hierarchically, but functions independently of Broca’s area (Varley et al., 2005). Friederici concludes “Broca’s area in the left hemisphere is crucial for the processing of hierarchy in language, but not for hierarchy processing in other higher-order cognitive domains, and can thus not be viewed as domain-general (p. 6).”

The structure of language, then, is based on the structure of thought, whether in the construction of episodes, the understanding of other people, the invention of music or mathematics, or sheer imagination. These various mental events are hierarchical, and lend their structure to their expression. As Pinker and Jackendoff (2005) remark, “the only reason language needs to be recursive is because its function is to express recursive thoughts (p. 230).”

## What of Other Species?

These considerations take the focus away from language as a communication system, and raises questions about the nature of thought itself and about the status of humans relative to other species. Are the hierarchical and generative aspects of thought truly uniquely human, and Berwick and Chomsky (2016) continue to insist, or can we find evidence for them prior to the emergence of humans, or indeed in contemporary non-human species?

For a start, it seems highly unlikely that they could have emerged in a single step in the promiscuous Prometheus a mere 100,000 or so years ago, as Chomsky has repeatedly claimed. Some 6–8 million years spanned the interval between modern humans and our common ancestry with apes (Langergraber et al., 2012), which is a more reasonable time period for the progressive evolution of hierarchical thinking. With respect to language itself, there are suggestions that at least some large-brained prehuman hominins, such as the Neanderthals, were fully verbally competent (e.g., Dediu and Levinson, 2013; Johansson, 2013; Hoffmann et al., 2018). But in the search for generative processes more generally, we can probably go back much further in evolution. Knott (2012) goes so far as to suggest that Chomsky’s Minimalist Program and the concept of Merge can be applied recursively to simple sensorimotor actions, such as grasping an object and bringing it to the mouth, activities common to primates species and seemingly intentional. This raises the question of whether Merge itself may have origins long predating language itself. Other aspects of animal action and thought also appear to exhibit at least a level of generativity comparable in kind to that in humans.

Many species, especially birds, do combine different signals. For example, Japanese tits have more than 10 different notes, and combine them to produce different warning signals (Suzuki et al., 2018). The three-note sequence ABC is a warning about predators, while another, D, is a call to attract conspecifics. The combined sequence, ABC-D, is a signal to recruit conspecifics to mob a stationary predator. There has been controversy as to whether this is genuinely combinatorial, retaining the meaning of each constituent, or whether each sequence is treated as a holistic unit (e.g., Bolhuis et al., 2010). At one level, at least, the combining of signals is an instance of Merge, but Merge itself can be considered to have different levels of recursion. Suzuki and Zuberbühler (2018) suggest four levels: 0, Merge, with no combination of element; 1, Merge, with combinations of elements but no recursion; 2, Merge, with merging of elements with previously merged combinations; and 3, Merge; with merging of different merged combinations. Recursive merging of the third type can generate unbounded hierarchical structures, or what Chomsky (1988) called “discrete infinity.” There is so far no evidence that any non-human species is capable of this level of Merge, or indeed of creating the vast number of meaningful utterances evident in human language. Even so, the idea of different levels of Merge suggests a degree of continuity rather than an abrupt saltation restricted to humans. And as suggested above, this level of generativity is better regarded as a property of thought, rather than of language itself.

## Mental Time Travel

Although it is commonly asserted that mental time travel itself is uniquely human (Tulving, 1985, 2002; Suddendorf and Corballis, 1997, 2007; Bulley et al., 2019), the evidence increasingly suggests that this is not true. It has long been known that honey bees perform waggle dances to indicate the location of food sources (von Frisch, 1967); even Hockett (1960) understood this to be an example of displacement, and it occurs not only in space but also in time (Plath and Barron, 2017). Evidence now also suggests that many vertebrate species have the capacity both to “replay” past events and imagine future ones (Corballis, 2013; but see Suddendorf, 2013). Some of the evidence is behavioral and comes from species as diverse as great apes (e.g., Martin-Ordas et al., 2010; Janmaat et al., 2014), birds (e.g., Clayton et al., 2003), rats (Wilson et al., 2013)., and even cuttlefish (Jozet-Alves et al., 2013).

In general, these studies suggest little of the generativity or expanse of human mental time travel. To be sure, there are prodigious feats of memory itself. The Clark’s nutcracker is said to cache some 33,000 seeds in around 7,000 locations every fall and relies on spatial memory to recover them over the winter (Kamil and Balda, 1985). The giant tortoise may not be a creative animal, but seems to have explicit memories lasting up to 9 years (Gutnik et al., 2019). Perhaps because non-human species have no expressive language and therefore cannot verbally describe their memories, we are apt to underestimate their capabilities. Whether these memories are genuinely episodic is perhaps open to question. The Clark’s nutcracker may simply remember where the seed are located, without any episodic memory of the act of caching itself.

Some recent studies of memory in vertebrates may offer more compelling evidence of human-like episodic memory for multiple events. In one study, rats remembered many different episodes over intervals of up to 45 min without any evidence of decline in performance (Panoz-Brown et al., 2016). Panoz-Brown et al. (2018) later showed that rats could remember different ordered sequences of odors associated with different contexts, implying memory for structured episodes; to rats, odors appear to be as distinctive and memorable as visual images are to humans. Accuracy was little affected by a delay of 60 min between encoding and testing, or by inserting an unrelated task, implying long-term episodic memory.

Other evidence for mental time travel comes from neurophysiology. Sequences of firing in “place cells” in the rat hippocampus not only track changes in location of the animal in a confined territory, such as a maze, but also track trajectories from past episodes in the maze, as well as possible future trajectories, or even purely exploratory ones. This hippocampal activity also records non-spatial associations tied to past events; the ability of rats to recall past sequences of odors was impaired following chemical suppression of hippocampal activity (Panoz-Brown et al., 2018). These observations appear to have the hallmarks of mental time travel, as though mentally “replaying” the past or imagining the future (Corballis, 2013; Moser et al., 2015).

There is perhaps still some doubt as to whether putative examples of mental time travel in non-human animals have the recursive structure of human imagination. Bulley et al. (2019) suggest that humans go beyond episodic projection to what they term *metaforesight*, the capacity to monitor, control and augment imagined futures, and argue that this superordinate capacity is unique to humans. Metaforesight is analogous to *metamemor*y, the comparable ability to monitor and control recollections of the past (e.g., Cavanaugh, 1982). Hence mental time travel, in humans, at least, may itself be under superordinate control, and hierarchically organized. Bulley et al. (2019) relate the emergence of Acheulian hand axes to dawning metaforesight from some 1.76 million years ago. Even so, we should perhaps not discount the seemingly free trajectories implied by hippocampal recordings in the rat as a form of controlled future planning. Pastalkova et al. (2008) showed that hippocampal recording could predict which way a rat plans to turn on the next trial in a maze, and suggest, “the neuronal algorithms, having evolved for the computation of distances, can also support the episodic recall of events and the planning of action sequences and goals (p. 1327).” Lewis et al. (2019) review evidence that great apes’ ability to spontaneously recall past events after long intervals is at least comparable to that in humans, again questioning human uniqueness, and implying a degree of superordinate control. The chimpanzee Panzee communicates with cards and keyboards, and in a typical study watched while researchers hid dozens of objects—fruits, toys, balloons, paper shapes—outside of his enclosure, and when later shown the symbol for each could guide a keeper to where it was hidden. Performance was accurate after 16 h (Menzel, 2005).

It may be true that mental travels, and imagination generally, are more profuse, flexible and “generative” in humans than in other animals. In the study by Martin et al. (2011), mentioned earlier, our human participants had little difficulty recapturing multiple past memories and imagining new scenarios. Such flexibility might also explain the human disposition for storytelling, once we evolved the capacity to externalize. In spite of our ability to bore listeners with seemingly endless exploits from the past, we probably actually remember only a small proportion of the vast number of events that punctuate our lives. It has been suggested that memory capacity may be partly sacrificed for flexibility itself (Tello-Ramos et al., 2019). For example, food-caching animals require extensive long-term memory for later retrieval of caches but show greater proactive interference, suggesting decreased flexibility, than in non-caching animals. In contrast, nomadic animals that move constantly to different environments may show great flexibility in acquiring new information, but also poorer retention of older information that is no longer relevant. On this analysis, humans may lie toward the extreme of high flexibility but relatively poor long-term retention.

This possibility is further elaborated by the historian Fernandez-Armesto (2019), who suggests that it underlies human creativity, and is yet another basis for human uniqueness, albeit at the expense of memory capacity:

The degree to which humans are, as far as we know, uniquely creative seems vast by comparison with any of the other ways in which we have traditionally been said to excel other animals (p. 3).

Claims of human uniqueness, though, run the risk of what has been called the “human superiority complex” (Villa and Roebroeks, 2014, p. 1) and the safer conclusion is that there are interspecies difference in the deployment of imagination. Claims of human uniqueness seem to progressively dwindle in the face of growing evidence for constructive thinking in non-human animals.

## Theory of Mind

Just over 40 years ago, Premack and Woodruff (1978) raised the question of whether our closest non-human relative were capable adopting the mental perspective of others. Thirty years later, opinion was still sharply divided. Penn et al. (2008) argued that the failure to recognize the mental discontinuity between animals and humans was “Darwin’s mistake,” while Call and Tomasello (2008) concluded that chimpanzees do have an understanding of the goals, intentions, perceptions, and knowledge of others, but no understanding of others’ beliefs or desires. Even ravens may show an understanding of what unseen birds can see Bugnyar et al. (2016). With respect to language, though, the critical question is whether an individual can understand what another individual *believes*. Over the succeeding decade, there has been some indication that great apes, at least, do have some understanding of what others believe.

The gold standard for assessing theory of mind at the level of belief is what has been termed the Sally-Anne test, designed to assess whether an individual understands that another individual has a false belief. In the original version, a child is shown two dolls, one called Sally and the other called Anne. Sally has a basket and Anne has a box. Sally puts a marble in her basket and leaves, and Anne then puts the marble in her box. Sally then comes back and the child is then asked where she will look for the marble. Autistic children say she will look in the box, but 4–6 year-old children understand that Sally has a false belief and say she will look in the basket (Baron-Cohen et al., 1985). Children aged four understand false belief but children aged three do not (Grosse et al., 2017).

Krupenye et al. (2016) tested three species of great apes (chimpanzees, bonobos and orangutans) on a version of the Sally-Anne test. A person hid an object and left, and the object was then moved. When the person came back, the animals look toward the original location, as though expecting the person to look there. That is, they behaved as though understanding that the person had a false belief. It is possible, though, that the apes had simply learned that people tend to look for things where they last saw them, and were not considering what the person believed. Kano et al. (2019) offer a more exacting test in which the apes saw a video in which an actor saw an object hidden under one of two boxes. The actor then moved behind a barrier that was either translucent or opaque, and the object was shifted to the other box. The eye movements of the apes were recorded, and only apes that had previously experienced the barrier as opaque visually anticipated that the actor would mistakenly look under the box where the object was originally hidden. That is, they were able to judge the actor’s belief based on their own past experience.

It may still be the case that theory of mind in apes is not at the level required for human language. An ape may know what another ape is thinking, but may not know that the other ape knows this, which Grice believed was required for meaningful discourse between the two. Again, though, we should be wary of the “human superiority complex” which seems to denigrate all attempts to demonstrate human-like intelligence in other species. And even if theory of mind is at a lower level in apes than in humans, we might still agree with Darwin (1871) that “[T]he difference in mind between man and the higher animals, great as it is, certainly is one of degree and not of kind” (p. 126).

## So What Is Special About Humans?

In spite of increasing evidence of cognitive continuity between humans and other species, we humans do seem exceptional in the ability to communicate, manufacture objects, and desecrate the planet. This difference may indeed relate to language, but perhaps less in the cognitive component than in the power of communication itself. This is counter to Chomsky’s proposal. To him, the communicative aspect is relatively trivial and uninteresting: “… externalization (hence a fortiori communication) is an ancillary aspect of language, peripheral to its core nature” (Chomsky, 2015a, p. 101).

I suggest here that it is more the communicative than the cognitive aspect that is largely responsible for our dominance on the planet. Imaginative thinking, whether directed to past or future events, or simply to the invention of scenarios, is no doubt adaptive to the individual, and may be common to many other species. What makes it special to humans is the ability to share it. This may be relatively trivial biologically, as Chomsky implies, but hugely important in our capacity to adapt to life on earth.

Most obviously, it simply increases the amount of information. The mental travels of others are incorporated into one’s own, albeit with some loss of precision and personal relevance, but with vast increase in scope. The contents of our mental lives are derived as much from others as from personal experience, probably more so. Large portions of our lives are spent in the imaginations of others. It is stories, whether in the form of fiction, soap operas, tales around the campfire, or gossip, that prompted Niles (2010) to rename our species *Homo narrans*—the storytellers.

Through sharing, communities know much more than the individuals within them, and can make that information accessible to all. Preliterate communities told stories that were repeated down the generations, accompanies by song and dance. With the invention of writing, storage of information could become more lasting and accurate, and shared even more widely. Through books, computers, and the internet, communicative sharing had progressively fewer bounds, either geographically or in storage capacity. My own memory and communicative reach now seem lodged mainly in my laptop and i-phone. This is due much more to cultural invention than to any innate disposition, although it remains variable across cultures. There remain indigenous peoples without these technological facilities who are fully endowed cognitively, and may well have retained cognitive abilities in excess of those who live in the modern industrialized state.

## The Production Problems

These advantages, though, raise the question of why other intelligent species have not evolved a comparable capacity to share. At least part of the answer has to do with the mechanisms of externalization, the production of signals that can meet the complexity of generative thought. Hippocampal recordings from the rat brain imply mental traveling well in excess of any signaling, vocal or otherwise, and it is now clear that many non-human species can comprehend much more than they can transmit. Bottlenosed dolphins easily learned human gestural signals instructing them to repeat up to 36 different behaviors, some of them complex and novel, but are themselves unable to make such gestures (Mercado et al., 1998). Two border collies appear to have receptive vocabularies in the hundreds (Kaminski et al., 2004; Pilley and Reid, 2011), but virtually no ability to articulate. Domestic dogs also recognize familiar speakers from their voice quality (Root-Gutteridge et al., 2019). Savage-Rumbaugh et al. (1998) reported that Kanzi, a bonobo, was able to follow simple spoken instructions, made up of several words, at a level comparable to that of 2.5-year-old child. Kanzi is now said to understand some 3,000 spoken words (Raffaele, 2006), but has virtually no ability to speak. Fischer and Hammerschmidt (2019) note that the structure of chimpanzee calls is largely innate, with only limited evidence for modification or conventionalization, while in contrast “comprehension learning may be extremely rapid and open-ended” (p. 1).

Vocal signaling seems especially constrained, especially in non-human primates. Control of the laryngeal muscles in the premotor cortex is only indirect (Simonyan and Horwitz, 2011; Koda et al., 2018), but well developed in humans. Koda et al. (2018) note, though, that even this is not sufficient for articulate speech, which also requires fine motor control of jaws, lips, tongue, and diaphragm—all of which constitute a “unique form of systems integration” (p. 11). These transformative changes presumably occurred sometime in the course of hominin evolution, well after the split from the great apes.

Vocal production, though, is not the only avenue for externalization. Manual action offers equivalent flexibility and intentionality, as is evident from the signed languages invented by deaf communities, and probably allows greater evolutionary continuity. In contrast to their poor voluntary control of vocalization, non-human primates are well adapted for intentional manual activity, whether in climbing, picking berries, grooming, or play. Attempts to teach great apes to talk have largely failed, but greater success has been achieved using simplified forms of sign language (Patterson and Gordon, 2001) or keyboards containing arrays of word-like symbols (Savage-Rumbaugh et al., 1998). Byrne et al. (2017) list 84 different communicative gestures arising from the studies of great apes’ gestures, and note that they are goal directed and intentional, unlike most primate calls. To be sure, there is little evidence for sentence-like structure, but chimpanzee gestures suggest a natural platform for more complex sequences.

These considerations have led many, including myself, to propose that productive language may have originated in manual gestures (e.g., Hewes, 1973; Fano, 1992; Armstrong et al., 1995; Rizzolatti and Arbib, 1998; Corballis, 2002, 2019; Arbib, 2005; Tomasello, 2008). In a recent study, children were asked to communicate about a picture in dyadic pairs, but denied the opportunity to speak. In less than 30 s, 4-to-6-year-old children developed systems of communicating in gestures, which rapidly became abstract and conventionalized. In 6- to 8-year old children, the gestures also showed evidence of grammatical structure. Gesture, then, seems as “natural” as speech (Bohn et al., 2019).

In evolutionary terms, gestures may have developed into pantomime, with increasing sequential properties. Although there is some limited evidence for pantomime in great apes (e.g., Boesch, 1993; Russon and Andrews, 2001), the critical period may have been the Pleistocene, dating from around 2.9 million to about 12,000 years ago, and heralding the emergence of the genus *Homo*. This era saw a tripling of brain size, obligate bipedalism, and the making of stone tools, and is also widely recognized as the period in which hominins established what has been termed the “cognitive niche” (Tooby and DeVore, 1987), establishing social bonding and enhanced communication for survival in the more exposed and dangerous environment of the African savanna. These developments probably established the setting for the emergence of pantomime as a dominant mode of intentional communication, enabling sharing of episodes or plans, perhaps resembling the modern game of charades. In the interests of efficiency, pantomime would become less iconic and more conventionalized by custom, perhaps to resemble modern sign language. Tomasello (2008), for example, writes of the possibility “that the human capacity evolved quite a long way in the service of gestural communication alone, and the vocal capacity is actually a very recent overlay (p. 246).”

The transition from gesture to speech was itself likely to have been gradual, with facial gestures accompanying manual ones (Corballis, 2017). Primates have intentional control over facial movements (Dobson and Sherwood, 2011) and Shepherd and Freiwald (2018) show that facial movements, such as the lip smack, act as visual signals in marmosets in so-called second-person social settings, involving interaction between signaler and audience. Production of these movements recruits areas homologous to Broca’s area in humans. Facial movements are also an important component of the sign languages, and people engaged in sign language watch face as much as they watch the hands, sometimes more so (Muir and Richardson, 2005).

In any event, speech itself is primarily gestural, based on movements of the lips, tongue, and larynx, but largely invisible to the viewer, so that sound was added to make them accessible. Indeed the retreat of gestures into the mouth can be regarded as part of the conventionalization process, and an early example of miniaturization (Corballis, 2017), although manual gestures remains an integral accompaniment to speech, even in the blind (Iverson and Goldin-Meadow, 1998). They can improve the speaker’s lexical access and fluency (Rauscher et al., 1996), and even reduce the speaker’s working memory load (Goldin-Meadow et al., 2001; Wagner et al., 2004).

The critical change that led to the development and vast expansion of communication options may have been the shift to obligate bipedalism. This freed the hands from locomotion, allowing them to play new roles in tool manufacture and communication; it may have been these growing demands that drove the shift to bipedalism itself. In anatomical terms, the change may have been relatively minor, but its consequences were immense. It is perhaps difficult to think of comparable changes with such dramatic consequences, but one possible example might be flight: One small step for bird, one giant leap for birdkind.

Communicative language therefore does seem to have a dramatic influence in human evolution, even if not in the way envisaged by Chomsky. But we may still exaggerate its benefits. As a sharing device, language is far from perfect; many complex thoughts or emotions seem to defy description. Albert Einstein is said to have developed the theory of relativity by imagining himself traveling on a light beam, and only with difficulty rendered it in mathematical terms. Often, too, it is more adaptive not to keep secrets and not have thoughts shared. In most non-human species, vocalization is largely involuntary, and acts as an “honest signal,” whereas language allows for deception, through lying and the dissemination of fake news. The 7,000 languages of the world are also testimony to the use of language as a moat, enabling sharing within groups but acting as a barrier between them—language seems to operate as much to prevent communication as to enable it. In many respects, then, language is an exclusionary and even destructive force. We should remember that at least 20 different species identified as hominins, but only humans survive, and have done so for only a few hundred thousand years.

## Some Conclusion

Macphail’s work captured something of the dilemma facing psychology in the latter part of the 20th century. Behavioral psychology was largely built on the commonalities of learning principles across different species, whereas the cognitive revolution was built on computational principles, with language as a primary exemplar supposedly unique to humans. The Rubicon seemed as impenetrable as it had seemed to Müller a century earlier.

In his later writing, though, Macphail was well aware that the science of animal behavior had moved on from behaviorism. Macphail and Bolhuis (2001), for example, wrote, “The behaviorist domination of experimental psychology had many unfortunate consequences, amongst them a divorce from those (now referred to as ethologists) who studied the behavior of animals” (p. 343). They go on to note that learning is often adapted to specific contexts. For example, rats seem especially adapted to learning about odors. Phobias, such as fear of snakes, seem to be learned much more rapidly than other forms of associative learning. Birds that store quantities of food, such as the afore-mentioned Clarke’s nutcracker, seem to have better spatial learning than non-storers, and indeed have larger hippocampi. Macphail and Bolhuis carefully review such examples, and conclude that there are no convincing examples of differences in the processes of learning and memory themselves, either within or between species. “The outcome,” they write, “supports a general process as opposed to an ecological account of cognition” (p. 361).

As noted at the beginning of this article, though, Macphail and Bolthuis continued to hold language as a special case, raising the intelligence of humans above that of any other species. Developments over the past few decades have seen a blurring of this gap. The essence of language seems to lie in generative thought rather than in any power of communication. This not only narrows the gap between humans and other species, but also broadens the concept of intelligence, which can now be taken to include theory of mind, imaginative thinking, and creativity. Macphail and Bolthuis do concede that they have omitted discussion of whether apes possess theory of mind, which Byrne (1995) had earlier suggested to be critical to the evolution of intelligence. As suggested earlier, we may add mental time travel and even imagination as aspects of intelligence that may go far back in evolution. We saw too that humans might sacrifice some memory capacity as a trade for enhanced creativity and imagination. It now seems likely that these capacities do vary even among vertebrates other than humans, contrary to Macphail’s suggestion that all non-human vertebrates are of equal intelligence. The picture seems complicated by suggestions of interactions between different capacities, as in the idea of a tradeoff between memory capacity and creativity. Given the advances in behavioral techniques and neurophysiological investigation, the challenge is to map out the mental capacities of different species, with perhaps less of an imperative to consider that humans are different.

This is not to undermine Macphail’s work and influence. Revisiting his work now reminds us that there was much value in the behaviorist movement that dominated psychology for much of the 20th century, establishing an evolutionary continuity that was largely overlooked following the “cognitive revolution.” He no doubt exaggerated the uniformity of intellect between species, at least if one overlooks language, but that kind of challenge spurs more critical research and better definitions of what is meant by intelligence. At the same time he did conform to the changing zeitgeist (with very little reference to Chomsky himself) emphasizing the special qualities possessed by humans, which may well have seeded the subsequent attempts to demonstrate cognition in other species, from birds to mammals (and perhaps not forgetting the worms). What may have seemed a stark contrast between humans and all other species has blended into a continuum, albeit one with added complexities and divergences. Macphail’s writing set up the challenge, and attempting to answer it can only advance our knowledge of how animals think, and where humans fit into the overall scheme of things.

## Author Contributions

The author confirms being the sole contributor of this work and has approved it for publication.

## Conflict of Interest

The author declares that the research was conducted in the absence of any commercial or financial relationships that could be construed as a potential conflict of interest.
